# ASPic-GeneID: A Lightweight Pipeline for Gene Prediction and Alternative Isoforms Detection

**DOI:** 10.1155/2013/502827

**Published:** 2013-11-07

**Authors:** Tyler Alioto, Ernesto Picardi, Roderic Guigó, Graziano Pesole

**Affiliations:** ^1^Centre Nacional d'Anàlisi Genòmica (CNAG), Parc Científic de Barcelona, 08028 Barcelona, Spain; ^2^Dipartimento di Bioscienze, Biotecnologie e Biofarmaceutica, Università degli Studi di Bari, 70126 Bari, Italy; ^3^Istituto di Biomembrane e Bioenergetica del Consiglio Nazionale delle Ricerche (CNR), 70126 Bari, Italy; ^4^Centre de Regulació Genòmica (CRG), 08003 Barcelona, Spain; ^5^Universitat Pompeu Fabra (UPF), 08003 Barcelona, Spain; ^6^Centro di Eccellenza in Genomica Comparata, Università degli Studi di Bari, 70126 Bari, Italy

## Abstract

New genomes are being sequenced at an increasingly rapid rate, far outpacing the rate at which manual gene annotation can be performed. Automated genome annotation is thus necessitated by this growth in genome projects; however, full-fledged annotation systems are usually home-grown and customized to a particular genome. There is thus a renewed need for accurate *ab initio* gene prediction methods. However, it is apparent that fully *ab initio* methods fall short of the required level of sensitivity and specificity for a quality annotation. Evidence in the form of expressed sequences gives the single biggest improvement in accuracy when used to inform gene predictions. Here, we present a lightweight pipeline for first-pass gene prediction on newly sequenced genomes. The two main components are ASPic, a program that derives highly accurate, albeit not necessarily complete, EST-based transcript annotations from EST alignments, and GeneID, a standard gene prediction program, which we have modified to take as evidence intron annotations. The introns output by ASPic CDS predictions is given to GeneID to constrain the exon-chaining process and produce predictions consistent with the underlying EST alignments. The pipeline was successfully tested on the entire *C. elegans* genome and the 44 ENCODE human pilot regions.

## 1. Introduction

Despite great efforts over the last ten years in computational gene prediction, translating a genome to a set of exon-intron structures and the proteins they encode is still a challenging task. The falling costs of traditional DNA sequencing and the development of next-generation sequencing technologies is leading to an accelerated number of complete genome sequences [1]. The sheer number of genomes sequenced argues for a real and continued need to design accurate computational tools for gene finding, the basic requirement being a first-pass set of reliable protein coding gene models [2].

Once the genomic sequence of a given organism has been completed, a common approach for annotating genes encoding proteins involves using *ab initio* or *de novo *gene prediction programs [2, 3]. *Ab initio* gene finders in fact allow quick and cost-effective analyses—a genome-wide set of vertebrate genes can be determined in only a few hours, for instance [4]. Many such programs are based on hidden Markov models (HMMs) and need to be trained before their application [2–7]. Capturing all gene features of an organism in a reduced training set is not a feasible task and thus, the accuracy of *ab initio* gene finders is mainly limited to the quality and size of the training set. Nonetheless, it is almost always that gene predictions obtained using *ab initio* systems represent the starting point for annotating newly sequenced genomes [2, 3].

Given the limited nature and accuracy of *ab initio *gene finders [8], new computational tools have been developed which take into account external evidence [2, 3, 9]. Methods based on comparative genomics have proven to be more accurate than previous systems even though they require that informant genomes be spaced at evolutionarily appropriate distances [10–12]. Newly sequenced genomes, however, do not always have an appropriately closely related genome available, reducing the global performances of such comparative methods. The recently sequenced grape genome [13], for instance, is not as strictly related to other available dicot plant genomes (such as *Arabidopsis thaliana *[14] or *Populus trichocarpa *[15] or *Lycopersicon esculentum *[16]) as those of human and mouse are to each other. However, during the last few years, methods using multiple genomes, which specifically take into account their evolutionary relationships, have been developed and are only beginning to show improvements over dual-genome prediction methods [11].

As emerged from the ENCODE Genome Annotation Assessment Project (EGASP) [8, 17], a community experiment to access the state of the art in genome annotation within the human ENCODE regions [18–20], programs relying on extrinsic evidence such as expressed sequence tags (ESTs) or mRNA sequences were found to be the most accurate in reproducing the manually curated annotations [8]. ESTs are in fact an invaluable source of evidence for the detection of exon-intron boundaries and likely alternative splicing variants [21]. Current methods for predicting genes using ESTs generally work by first performing an alignment of expressed sequence tags onto a target genomic region and then combining the alignment results with *ab initio* gene predictions [22]. However, the inclusion of EST alignments into HMM-based systems is not a simple task due to the requirement that alignments must be incorporated into the model in a probabilistic way, often leading to only negligible performance gains. A new version of HMMGene using EST evidence, for example, reported no improvement in predictions for *Drosophila melanogaster* [23]. Only methods combining EST alignments and comparative genomics such as TWINSCAN_EST or N_SCAN_EST and the recent Pairagon-N_SCAN_EST proved to be the most accurate in predicting exact exon-intron structures [8, 11, 24]. However, apart the from availability of one or more informant genomes, their approach to integrate information from EST alignments needs a training step. Also in this case, the quality and size of the training set may reduce the benefit due to ESTs, especially when they are used to predict genes in novel genomes with a limited amount of expression data. These limitations have been partially avoided by methods that use EST alignments to simulate the manual annotation. Exogean, for instance, is a program appropriately designed to employ EST or mRNA alignments as biological objects in a directed acyclic colored multigraphs (DACMs) [25]. Although Exogean has been indicated as one of the most accurate programs in predicting correct coding genes in EGASP project [8], it is subjected to strong limitations. A reduced number of available ESTs in fact may preclude gene prediction in genomic regions not covered by expression data.

In light of what has been previously discussed, we report here a simple and accurate method called ASPic-GeneID to improve gene prediction while maximizing the information gained from expressed sequence tags. Alignments of EST sequences to the genome are particularly good at pinpointing the location of splice sites and intronic sequence. Such introns can be easily used as evidence to improve the chaining of *ab initio* predicted exons, thus making gene models more accurate. Our procedure does not require complex probabilistic models and it is completely independent of EST training sets. Intronic sequences are directly inferred from expression data by means of the program ASPic [26, 27], whereas both the *ab initio* exon predictions and the gene assembly are performed using the GeneID software [28, 29]. Since ASPic is also able to detect the most likely transcript variants for a gene, we propose here two simple extensions to ASPic-GeneID that allow the prediction of alternative splicing transcripts.

We have tested ASPic-GeneID on the entire *C. elegans* genome (WS147) and the 44 human ENCODE regions and compared the results to those of programs representing the state of the art in nematode and human gene prediction. Our results suggest that ASPic-GeneID is a real and practical alternative to very complex pipelines that currently require all available evidence to obtain the same values of specificity and sensitivity.

In the next section, we explain in detail the methodology behind ASPic-GeneID and its implementation. Finally, we focus on ASPic-GeneID predictions on the human ENCODE regions and on the *C. elegans* genome.

## 2. Materials and Methods

### 2.1. The ASPic-GeneID Pipeline

ASPic-GeneID represents the integration of two complementary methods for predicting gene structures in a target genome: the *ab initio* gene predictor GeneID [28], and the alternative splicing prediction program ASPic [26, 27]. Given intronic locations deduced from alignment of expressed sequence tags (ESTs) to the genome, ASPic-GeneID attempts to predict complete gene structures in the target genome sequence.

The first component of the pipeline is ASPic, a method to predict alternative splicing isoforms expressed by a gene and their exon-intron organization at the genomic level through the information provided by available expression data, mostly EST sequences [26, 27]. In contrast with the majority of other tools for the analysis of alternative splicing, ASPic performs a multiple alignment of transcript data to the genomic sequence and refines exon-intron boundary alignments through dynamic programming [27]. Such techniques improve the quality of the splice site predictions by minimizing the number of false positives. ASPic also provides the minimal set of nonmergeable transcript variants compatible with the detected splicing events [27].

The other component of the pipeline is GeneID, a well-known *ab initio* gene finder that predicts and scores all potential coding exons along a query genomic sequence [28]. From the set of predicted exons, GeneID assembles the gene structure maximizing the sum of the scores of the assembled exons using a dynamic programming chaining algorithm [28]. The hierarchical structure of the program separates the problem of exon assembly from the prediction of coding exons along a given query genomic sequence. Simple rules describing the relationships among initial, internal, and terminal exons as well as other gene signals (poly-adenylation, etc.) have to be imposed in an appropriate and organism-specific external parameter file in order to infer the most likely gene structures [28].

Current *ab initio* gene finders, including GeneID, suffer from both low specificity (they tend to predict too many genes and exons) and less than perfect sensitivity (correct exons may be predicted with low scores and consequently excluded from the final gene structures), leading to inaccurate predictions.

**Figure 1:**
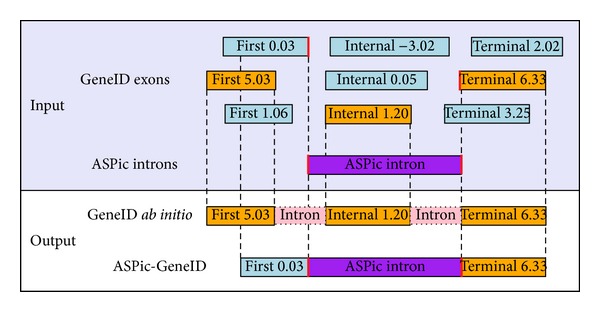
Graphical overview of ASPic-GeneID. In the absence of ASPic introns, the dynamic programming algorithm implemented in GeneID (called Genamic) assembles the most likely gene structure according to frame-compatible exons with the highest combined score. When ASPic introns are provided, they act as anchors in the chaining of exons so that exons with intron-compatible splice sites are always joined together if they conform to a valid gene model. In this hypothetical example, the “First” exon with the highest score (in orange) is replaced by the one with the lowest score (in blue), but which possesses an ASPic intron-compatible splice site (in red).

To improve both sensitivity and specificity of exon/transcript prediction, our novel procedure implemented in ASPic-GeneID is designed to improve the chaining of inferred GeneID exons by introducing constraints during gene assembly. We surmised that a good constraint candidate would be introns with reliable splice sites such as those predicted by ASPic. ASPic introns, in fact, are directly deduced from expression data and, thus, they constitute an invaluable source of evidence. We have introduced changes in GeneID so that the optimal path through the dynamic programming matrix is one which maximizes the number ASPic-inferred introns incorporated into the predicted transcript models. In other words, given a set of evidence introns, GeneID tries to join potential exons that have splice sites compatible with these introns (Figure 1).

### 2.2. Running ASPic-GeneID

Given a query genomic sequence (whether it is a single gene or a chromosome or a complete genome) and a collection of EST and/or mRNA sequences belonging to the same organism, we map all expressed sequences to the query sequence using GMAP [30], a computational tool specifically designed to reliably align a large number of ESTs and mRNAs to a genomic sequence. It has been shown that GMAP outperforms BLAT, which is another program widely used for the same purpose [30, 31]. The corresponding software has been downloaded from the website of the author (http://research-pub.gene.com/gmap/) and run with default parameters. Results of GMAP are then parsed to obtain clusters of ESTs and/or mRNAs related to specific regions of the query sequence. During the parsing only alignments with a minimum identity of 95% (98% in human) and EST coverage greater than 90% are retained. Each EST cluster should correspond to a specific gene. However, we may expect that different genes, depending on their peculiar expression profile are represented by clusters of different sizes or are not represented at all.

To construct clusters, we first collect overlapping ESTs and/or mRNAs according to GMAP coordinates on the genomic sequence and then we address compatible ESTs to the same cluster. Two ESTs or mRNAs are assumed to be compatible if they have at least one splice site in common, allowing a minimal mismatch around exon-intron boundaries in order to overcome potential GMAP misalignments or EST sequencing errors. In the case of unspliced ESTs, they are added to the relevant cluster according to mapping coordinates and strand. Each EST cluster and the mapping genomic sequence, form the input used by ASPic, run with default parameters, to predict introns. Depending on the coverage of the gene region by EST sequences, ASPic also provides a more or less reliable prediction of potential alternative transcripts.

After each ASPic run, we parse the corresponding output in order to collect all predicted introns in the general feature format [32]. The intron evidence is then given to GeneID which then predicts the most likely gene structures given this evidence and its statistical models for signals and coding sequence. The source code of GeneID has been updated in order to incorporate GeneID into the ASPic-GeneID context, in particular to accommodate the use of introns as evidence. A small adjustment has also been made to the parameter file in order to add introns to the gene model for the dynamic programming module implemented in GeneID.

We have written simple Python and Perl scripts to perform all the components of the ASPic-GeneID analysis transparently: the parsing of GMAP results, the generation of ESTs clusters, the ASPic intron predictions, and finally the GeneID predictions.

ASPic-GeneID has essentially no limits with respect to the length of the input sequence or the number of related ESTs. It can handle chromosomes as well as complete genomes.

### 2.3. Implementing the Alternative Splicing Prediction

The running of ASPic-GeneID as previously described allows the prediction of only one transcript per gene locus. Although this limitation should not reduce the gene prediction accuracy of our system in genomes with a low prevalence of alternative splicing, it is expected to affect the global performance in the case of genomes from organisms in which alternative splicing is a widespread phenomenon.

To overcome this limitation, we have implemented two extensions of ASPic-GeneID which allow for the prediction of alternative transcripts. In the first procedure, which we call ASPic-GeneID_AS1, we map ESTs to a query sequence using GMAP and then build EST clusters related to specific gene regions using exactly the same methodology as above. To each cluster and gene region we apply ASPic and collect in two separate files in GFF format all inferred introns and transcripts. Introns are used as evidence to run ASPic-GeneID as previously described and to obtain an initial gene set without alternative splicing. After that, all predicted ASPic-GeneID genes that overlap transcripts deduced by ASPic are removed. The remaining genes are then combined with transcripts inferred by ASPic to produce the final gene set. In this way, we employ ASPic alternative transcript predictions for all genomic regions fully covered by ESTs and/or mRNAs and ASPic-GeneID predictions for the remaining genomic regions partially covered or not covered by expression data.

In the second procedure, which we call ASPic-GeneID_AS2, we again map expression data to the query sequence using GMAP and run ASPic on each EST/mRNA cluster to collect deduced introns and full-length transcripts in GFF format. From each ASPic transcript we extract the longest open reading frame. Overlapping ASPic CDSs are then assigned to separate bins, where the number of bins used corresponds to the number transcripts belonging to the locus with the highest number of alternative transcripts. In order to maximally cover the genome, for loci with fewer transcripts than there are bins, ASPic CDS spans are reassigned to empty bins. We then run GeneID on each bin using both ASPic CDS and unassociated introns as evidence. Finally, we remove redundant identical transcripts from the combined predictions of each run of GeneID to produce a final gene set.

**Figure 2:**
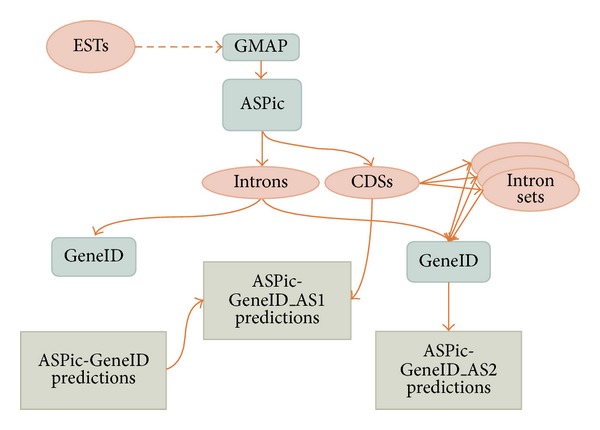
Relationship between ASPIC and GeneID predictions. ASPIC predicts both introns and full-length transcripts. When ASPIC introns are given to GeneID, we obtain ASPIC-GeneID predictions without alternative splicing. In contrast, when ASPIC transcripts are used, we can predict alternative variants in two ways. The first combining ASPIC transcripts and nonoverlapping ASPIC-GeneID predictions and the second giving ASPIC transcripts to GeneID as evidence and then removing redundant predictions.

Relationships between ASPic and GeneID are shown in Figure 2. When GeneID uses only ASPic introns we have ASPic-GeneID predictions without alternative splicing. In contrast, when ASPic transcripts are used in combination with ASPic-GeneID we have ASPic-GeneID_AS1 and ASPic-GeneID_AS2 predictions with alternative splicing (Figure 2).

### 2.4. Sequence and Prediction Sets

The *C. elegans* genome sequence version WS147 was downloaded from the WormBase website (http://www.wormbase.org/). Gene predictions from several other *ab initio* gene finders such as Genefinder (release 980504; P. Green, unpublished), FGENESH, and SNAP were downloaded from the Sanger Centre (http://www.sanger.ac.uk/Software/analysis/genomix/). TWINSCAN and TWINSCAN_EST predictions were downloaded from http://mblab.wustl.edu/. All available *C. elegans* ESTs and mRNAs have been retrieved from the Unigene database. We use Unigene sequences instead of dbEST sequences because Unigene sequences are filtered to avoid redundant and erroneous ESTs.

All 44 human ENCODE regions were downloaded from the UCSC genome browser (http://genome.ucsc.edu/) according to the human genome assembly hg17. Predictions from diverse gene finding programs belonging to different EGASP categories (*ab initio*, ESTs, mRNAs, and proteins based, all evidence based) were downloaded from the official EGASP repository (http://genome.imim.es/datasets/egasp2005/). The complete list of programs used in the evaluation is available in Table 2.

All human ESTs and mRNAs related to the 44 ENCODE regions were downloaded from GenBank according to their accession numbers retrieved from the Otter database [33].

### 2.5. Evaluation

Annotated *C. elegans* CDSs (WS147) were downloaded from WormBase. ASPic-GeneID predictions (including those from ASPic-GeneID_AS1 and ASPic-GeneID_AS2) as well as other predictions from different gene finding systems were evaluated against the annotation using an evaluation program written in Perl (Eduardo Eyras, personal communication), which takes into account alternative transcripts.

Briefly, the evaluation.pl program compares predictions and annotations in two ways: on a per gene basis and on a per best transcript pair (BTP) basis. For both methods, a gene is defined as a cluster of transcripts according to exon-overlap. For evaluation on the basis of a gene, the program performs a projection of all transcripts to the genome and then calculates for exons, introns, and nucleotides the sensitivity (SN), the specificity [13], the wrong cases (W), and the missing cases (M). All accuracy measures follow the definitions of Burset and Guigó [34]. Briefly, for each level (nucleotide, exon, and gene) the sensitivity is SN = TP/(TP + FN) and the specificity is SP = TN/(TN + FP), where TP are true positives, TN are true negatives, FN are false negatives, and FP are false positives [34].

Calculation of statistics on a BTP basis is performed as follows. For each transcript cluster, the evaluation program establishes a one-to-one (and one-to-many in the case of split/joined transcripts) mapping between predicted and annotated transcripts. It then produces similar measures as above but only for best transcript pairs. These measures give a better estimate of the accuracy of connectivity of the predicted transcripts.

Summary statistics for both methods given in the results are derived from total feature counts for the entire evaluation set.

The accuracy of ASPic-GeneID has been evaluated on two different data sets, the entire *C. elegans* genome (version WS147), and the 44 human ENCODE pilot regions, using WormBase and Gencode annotations, respectively, as the reference annotations. Such data sets have been appropriately chosen to better assess the performances of ASPic-GeneID in two organisms differentially subjected to alternative splicing. Moreover, *C. elegans* and human ENCODE regions differ in the amount of EST coverage, which in turn can affect the quality of ASPic predictions. In the ENCODE pilot regions, the EST coverage is nearly complete: 91.5% of all introns (98.6% of introns in coding sequence) are covered by ESTs at a specificity of 85%. In contrast, ESTs cover only about 60% of the *C. elegans* genome which often results in EST clusters with incomplete exon-intron structures.

In the case of the *C. elegans* genome, we have compared ASPic-GeneID predictions with those from programs representing the state of the art in nematode gene finding, including *ab initio*, comparative, and EST-based methods (Table 1). Likewise, all predictions on the 44 ENCODE regions have been compared to those from a number of established gene finding programs involved in the human ENCODE genome annotation assessment project (EGASP) (Table 2).

### 2.6. Availability

GeneID source code (version 1.3) as well as *C. elegans* and human parameter files can be downloaded from http://genome.crg.es/software/geneid/. For large-scale jobs, we recommend to download the off-line version of ASPic from the following web page: http://150.145.82.212/aspic/aspicgeneid.tar.gz. In addition, Python and Perl scripts to automate ASPic are also provided (including all ESTs that could get a very big file. On the other hand, ESTs sequences can easily be downloaded from Unigene database).

Additional Python and Perl scripts to automate ASPic-GeneID (for Linux and Mac OS X) are available upon request.

## 3. Results and Discussion

### 3.1. ASPic Intron and Gene Prediction in *C. elegans* Genome and All 44 ENCODE Regions

The underlying principle of our system is that introns can guide *ab initio* gene assembly. This task, however, can only be addressed using reliably predicted introns. Available methods to align EST sequences to the genome are mainly based on BLAST [35] or BLAT [31] and sometimes lead to poor splice site predictions. In order to obtain a high-quality set of intron positions, we first mapped all available *C. elegans* ESTs onto the complete worm genome (version WS147) using GMAP as described in Section 2. Then, EST clusters related to potential gene regions were exploited to run ASPic. In contrast to other EST to genome alignment programs, ASPic employs a novel and efficient algorithm to minimize the number of exon predictions and hence of alignment inferred splice sites. ASPic is also able to infer alternative splicing variants of a gene given a related collection of ESTs.

When applied to each *C. elegans* EST cluster, ASPic can predict intron sequences and also full-length splicing variants whenever ESTs completely cover specific gene regions.

In all six *C. elegans* chromosomes, ASPic proves to be extremely specific. Out of 100723 predicted introns, 96.1% exactly match (with the same exact splice sites) annotated introns. However, overall sensitivity is low—likely due to the fact that coverage of the genome by ESTs is only about 60%. 

Overall, ASPic's nucleotide and exon specificities are 98% and 91%, respectively. ASPic is also very specific when comparing only to the best overlapped transcripts where nucleotide and exon specificities increase to 99% and 95%, respectively.

Moreover, it is able to find exact transcript variants with a specificity of 71%, which is the highest reported up to now. Similar specificity values have been reported by Genomix, a new gene finder system working as a combiner [36]. However, Genomix specificities at exon and nucleotide levels of 87.3% and 91.9%, respectively, have been calculated only on a reduced subset of 1534 confirmed *C. elegans* genes (version WS147) [36].

Despite the high specificity, ASPic shows very low sensitivity values at all levels except when the comparison with the annotation is limited to only transcripts overlapped by a prediction (BTP level). In this case, the accuracy of ASPic, as given by (Sn + Sp)/2, at the nucleotide and exon levels are 90% and 88%, respectively.

It has been currently demonstrated that the expected distribution of spliceosomal intron lengths is correlated to the quality of the annotation [37]. Since introns are removed after transcription, intron lengths are not expected to respect coding frame. For this reason, the number of genomic introns that are multiple of three bases should be similar to the number of introns that are a multiple of three plus one or two bases [37]. In effect, ASPic predicted introns follow this behaviour. Of all *elegans* inferred introns, 33.5% are a multiple of three bases, whereas 33.6% and 33.0% are multiples of three plus one and two bases, respectively. These results strongly corroborate ASPic's ability to predict *bona fide* exon-intron boundaries.

The same approach used for the complete *C. elegans* genome has been applied to all 44 ENCODE human regions. In this case, however, single EST clusters related to gene regions have been generated using a subset of all available human expressed sequence tags downloaded from the Otter database in order to reduce potential pitfalls due to low quality ESTs or to aberrant mRNAs from pathological tissues.

ASPic is able to predict more introns than annotated in ENCODE. However, we focus only on annotated coding regions and it is well known that ENCODE contains many noncoding transcripts in addition to a number of introns located in UTR regions. Restricting, thus, the comparison to coding regions only, we found ASPic to be the most accurate system to predict introns in human ENCODE. This is derived mostly from its higher specificity; it is the most specific, with 87% of all predicted introns corresponding exactly to an annotated intron. This value increases to 96% when making the BTP comparison, demonstrating that the novel alignment algorithm behind ASPic is quite efficient and results can be comparable to those based on PAIRAGON, indicated as one of the best program to align mRNA sequences to genome [24, 38]. As shown in Table 2, ASPic outperforms PAIRAGON-any in predicting correct introns. Considering that PAIRAGON-any aligns only high quality sequences from the full ORF Mammalian Gene Collection (MGC) [39] and from the human RefSeq database, ASPic's performance which is based only on ESTs is even more remarkable.

ASPic is not highly specific at the transcript level where it is outperformed by Exogean and PAIRAGON-any. However, it is as specific as the combiner Fgenesh++ [40] and it is 12% more sensitive at the transcript level than Exogean and PAIRAGON-any. ASPic is also more sensitive than Ensembl [41], AUGUSTUS-any, and AUGUSTUS-EST at the exon level [22]. When comparing at the BPT level, it has the highest exon specificity (95%) (Table 2).

Like for the previous results described for the whole *C. elegans* genome, in the human ENCODE regions, ASPic predicted intron length distributions are not skewed. Of all ASPic introns, 33.0% are a multiple of three bases and 33.2% and 33.7% are multiples of three plus one and two bases, respectively.

### 3.2. ASPic-GeneID Accuracy without Alternative Splicing

Depending on the EST coverage of each gene region, ASPic can predict just introns or both introns and alternative splicing variants. For this reason, we can independently use two main sources of evidence from ESTs such as individual introns and full-length transcripts to improve GeneID *ab initio* predictions. When only introns are given as evidence to GeneID, the program is able to predict at most one transcript per locus. As outlined in Section 2, introns with correct splice sites can aid the correct assembly of *ab initio* predicted exons during the exon-chaining step. The dynamic programming procedure implemented in GeneID builds gene structures using exons with the highest scores respecting frame compatibility and gene model rules [28]. The introduction of ASPic introns to GeneID forces exons with compatible frames and splice sites to be joined. Since such evidence introns do not interfere with the main GeneID exon prediction process, it is expected that they are used only when compatible *ab initio* exons really exist. Our procedure to handle evidence introns as implemented in ASPic-GeneID is also expected to improve the accuracy at the transcript level.

When all ASPic predicted introns on the complete *C. elegans* genome are given as evidence to GeneID, our combined ASPic-GeneID system is found to be the most accurate in predicting exact nematode transcripts. The results show 21% improvement in sensitivity and 24% in specificity in predicting exact transcript structures compared to GeneID, which does not use ASPic introns (Table 1). ASPic-GeneID is, in turn, significantly more accurate than SNAP [4], FGENESH [42], and GENEFINDER [8], the most widely used *ab initio* gene prediction program for nematodes. Moreover, ASPic-GeneID outperforms TWINSCAN [43], which uses the *C. briggsae* genome as an informant genome, at all sensitivity and specificity measures. Most interestingly, our gene prediction method is also more accurate than TWINSCAN_EST [9], a new system that combines EST alignments with TWINSCAN. In particular, ASPic-GeneID is 6% more sensitive at the exon level than TWINSCAN_EST. Taking the mean between sensitivity and specificity, ASPic-GeneID is also more accurate than TWINSCAN_EST in predicting exact transcript structures, 45.5% versus 44%.

**Figure 3:**
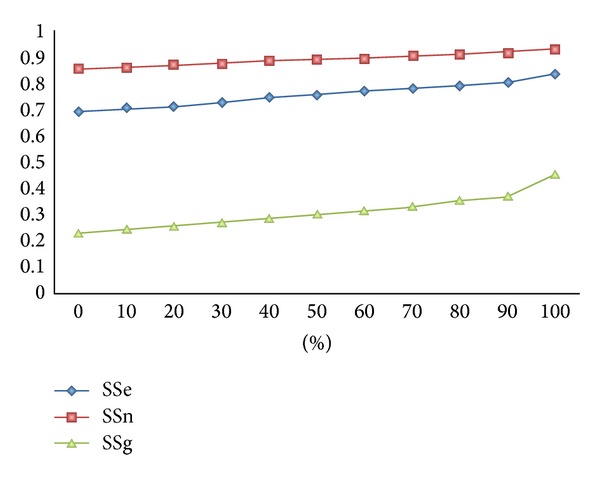
ASPic-GeneID performance on *C. elegans* according to number of introns. The accuracy [(Sn + Sp)/2] of ASPic-GeneID predictions (AG) is plotted according to the proportion of introns output by ASPic and provided to GeneID as evidence. SSe, SSn, and SSg indicate the accuracy at exon, nucleotide, and gene level, respectively.

The strength of ASPic-GeneID relies on the use of reliable intron sequences. Even when EST genome coverage is not high and, thus, the number of ASPic predicted introns is low, our system should predict genes with an accuracy better than GeneID alone. To verify the effect of the number of introns in improving *ab initio* GeneID predictions, we performed the following experiment. ASPic was run on the complete *C. elegans* genome using all available ESTs as described in Section 2. Then, from all the predicted introns we randomly selected increasing percentages of introns ranging from 0% to 100% and ran GeneID using each intron subset. The number of introns is undoubtedly related to the EST genome coverage and, thus, a low number of ESTs should yield a low number of introns. Results of this experiment are given in Figure 3 where the averages between sensitivity and specificity at gene [44], exon (SSe), and nucleotide levels (SSn) are reported as a function of growing intron percentages. The benefit due to introns increases linearly with the number of input introns and we can register a gene prediction improvement at all levels, even when the number of introns is very low (10%). These data indicate that ESTs and, thus, introns related to some genes can improve the accuracy of neighbouring genes. In practice, GeneID mistakes such as extension and inclusion of exons in neighbour genes become much less common because introns introduce real constraints in gene assembly.

The accuracy of ASPic-GeneID using only introns has also been evaluated on all 44 human ENCODE regions. Here, however, the situation is quite different because human genes are subjected to extensive alternative splicing and because human gene density is low. A system such as ASPic-GeneID which predicts only one transcript per locus is a disadvantage. Nonetheless, ASPic-GeneID outperforms all *ab initio* gene prediction programs such as Genemark [45] or AUGUSTUS_abinitio [5], currently one of the most accurate programs to find *ab initio* gene structures in mammals [8]. ASPic-GeneID is 18% more sensitive and specific than GeneID alone in predicting exact transcript structures. Moreover, ASPic-GeneID accuracy at the exon level is 73%, a value which is higher than the corresponding value obtained from other systems that use ESTs such as ExonHunter [46] or informant genomes such as TWINSCAN [43], SGP2 (an extension of GeneID) [10], DOGFISH [8], and AUGUSTUS_dual [22] or both evidence sources such as FEGENESH++ [40]. However, our system is less accurate at exon and nucleotide level than programs that use all available evidence for human (Ensembl, PAIRAGON-any, and AUGUSTUS-any) or programs that predict more than one transcript per locus (Exogean [25]). Nonetheless, in several measures, ASPic-GeneID shows high sensitivity. At the exon level, for instance, ASPic-GeneID sensitivity is 81%, 1% more than AUGUSTUS_EST, an improved version of AUGUSTUS that uses ESTs and proteins alignments, 6% more than PAIRAGON_multiple and 3% more than FGENESH++, a combiner that uses all available evidence. In the BTP comparison, ASPic-GeneID sensitivity at the exon level increases to 86%, 2% higher than Ensembl.

On the whole, ASPic-GeneID remains one of most accurate systems to predict correct intronic sequences, attesting its sensitivity at 86% and specificity at 72%. These last values go up to 91% and 81%, respectively, when intron evaluation is assessed at the BTP level. 

**Figure 4:**
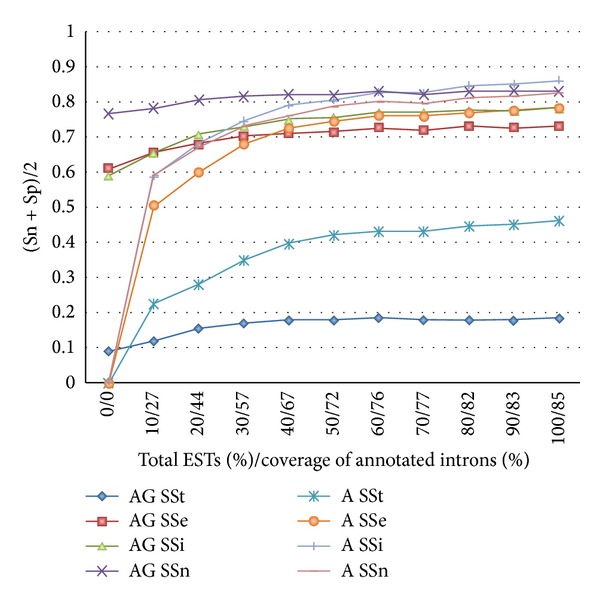
ASPic and ASPic-GeneID performance on ENCODE according to EST coverage. The accuracy [(Sn + Sp)/2] of ASPic CDS predictions (A) and ASPic-GeneID predictions (AG) is plotted according to the proportion of available ESTs given as input to ASPic. The percent coverage of annotated introns by the input ESTs is also given. SSn, SSe, SSi, and SSt indicate the accuracy at nucleotide, exon, intron, and transcript level, respectively.

We noted, however, that ASPic CDS predictions alone are better than those of the combined ASPic-GeneID on the ENCODE regions (see Figure 4). We surmised that this must be due to EST coverage. Unlike in *C. elegans*, where EST coverage is somewhat low, the coverage of annotated human coding sequences by human ESTs is very high (85% of all introns and nearly 99% of introns in coding sequences). To determine at what level of EST coverage using our combined approach may be beneficial, we performed the following experiment. We selected random sets of ESTs corresponding to 10%, 20%, 30%, and so forth up to 100% of the ESTs available as input to ASPic. These EST sets had an intron coverage ranging from 27% to 85% of annotated introns. When using less than 35% of the available ESTs (corresponding to about 62% intron coverage) ASPic-GeneID performed better at the exon level than ASPic alone. At higher coverage, we found that ASPic CDS predictions are clearly more accurate. The performance of ASPic at the transcript level is quite good even at low EST coverage levels. This is perhaps due to the presence of a class of highly expressed transcripts that are well covered by ESTs. ASPic will predict them correctly, while ASPic-GeneID may try to extend the transcripts with additional predicted exons.

### 3.3. ASPic-GeneID Accuracy with Alternative Splicing

In order to improve the performance of ASPic-GeneID, especially in mammalian gene finding, we implemented the possibility to predict alternative transcripts. In particular, we addressed the alternative splicing task with two independent procedures that should be considered simple extensions of ASPic-GeneID. In the first procedure, we predicted alternative variants by ASPic in genomic regions fully covered by ESTs and then we added all ASPic-GeneID predictions not overlapping ASPic transcripts. This procedure, called here ASPic-GeneID_AS1, combines in the simplest manner ASPic and ASPic-GeneID predictions, giving rise to ASPic predictions because they are directly deduced by expression data. The second procedure, called here ASPic-GeneID_AS2, is, instead, mainly dependent on GeneID. As for ASPic-GeneID_AS1, a complete pool of alternative transcripts was obtained by ASPic. Each predicted variant (represented by a set of exon-linked introns) was then given to GeneID as evidence. All predicted transcripts were finally combined and filtered in order to produce the final nonredundant set of transcript predictions (more details in Section 2).

Overall, results from ASPic-GeneID_AS1 and ASPic-GeneID_AS2 on the 44 ENCODE human regions are quite similar and overlapping (Table 2). Both procedures outperform ASPic-GeneID at all levels in the BTP comparison. Although ASPic-GeneID_AS1 is not more sensitive than ASPic-GeneID_AS2, it appears to be more specific (as it directly utilizes all ASPic transcripts). As shown in Table 2, all results can be compared to those from programs that currently use all available evidence or protein alignments to improve gene prediction in human.

In particular, when predictions are evaluated at gene level, ASPic-GeneID_AS2 is the most sensitive in finding genes (96%), exons (90%), introns (90%), and nucleotides (93%). In contrast, ASPic-GeneID_AS1 is the most specific at the BTP exon, intron and nucleotide levels. Focusing on methods using ESTs and mRNAs alignments but excluding proteins, ASPic-GeneID_AS1 is as accurate as PAIRAGON-any (49%) and 2% more accurate than Exogean (47%), indicated as the best gene finding program by the EGASP assessment, in predicting exact transcript structures. Moreover, ASPic-GeneID_AS1 has a transcript sensitivity of 65% which is the highest registered up to now. In the comparison with one of the most widely used pipelines as Ensembl, ASPic-GeneID_AS1 is 2% more accurate at both exon and intron levels. On the other hand, at transcript level both our systems are on average 22% and 3.5% more accurate than Ensembl in finding transcripts and exons, respectively.

On the whole, as shown in Table 2, ASPic-GeneID_AS1 and ASPic-GeneID_AS2 appear to outperform also many other well-established gene prediction tools at different measures. Although it is difficult to assess which program is really the best annotation system for human ENCODE regions, our simple methods that use only ESTs as main source of evidence prove highly competitive and comparable to very complex pipelines.

When we move to the *C. elegans* genome in which the impact of alternative splicing is low, the performances of ASPic-GeneID_AS1 and ASPic-GeneID_AS2 are slightly better than ASPic-GeneID at all levels. However, the possibility to predict alternative transcripts improves the global finding of exact transcripts and exons. At the gene level ASPic-GeneID_AS2 seems to be more accurate than ASPic-GeneID_AS1. In contrast, at transcript level, ASPic-GeneID_AS1 appears to be more efficient than ASPic-GeneID_AS2 since it directly uses ASPic inferred transcripts.

### 3.4. ASPic-GeneID and Deep Transcriptome Sequencing

ASPic-GeneID has been developed to handle long transcriptome sequences as main biological evidence to improve gene structures and detect potential alternative splicing transcripts.

Current high-throughput sequencing methodologies as RNA-Seq aim to provide global overview of entire transcriptomes. However, huge amount of short reads from Illumina or SOLiD platforms pose other challenges than classical Sanger ESTs and in many cases the detection of reliable transcripts is not optimal. Long-reads, therefore, as those from Sanger sequencing or the Roche 454 sequencer (Titanium chemistry with reads longer than 500 bases) represent the main source of evidence to reliably identify splice sites and alternative isoforms, other than simplify the deciphering of complex eukaryotic gene structures. 

ASPic-GeneID is ready to analyse long EST-like reads from modern sequencer as Roche 454 and very long reads that are coming with the third generation sequencing platforms as PacBio. Although ESTs and ESTs-like sequences are optimal for our pipeline, in principle it could work with Illumina reads. However, computational times are expected to be very onerous and no extensive tests have been performed to assess the biological quality of results.

## 4. Conclusions

Despite the advent of novel sequencing technologies [47, 48], the accurate genome annotation is yet a hard and challenging task. In order to improve such a process once a new genome sequence has been completed, we proposed here a simple computational strategy to accurately identify coding regions employing expressed sequences, mostly ESTs. Our framework, called Aspic-GeneID, uses EST based predictions by Aspic to improve *ab initio* gene structures by GeneID. Moreover, it can predict alternative transcripts providing a global view of the transcriptome. Aspic-GeneID is quite flexible depending on EST coverage. In organisms with a low impact of alternative splicing as *C. elegans*, it provides optimal predictions resulting in one of the most accurate gene finding programs. In contrast, when the impact of alternative splicing is high as in human, it can outperform existing gene finders at different levels. Moreover, the ability to predict multiple transcripts per gene locus makes Aspic-GeneID results comparable with those from very complicated pipelines like Ensembl, PAIRAGON-any, or AUGUSTUS-any that tend to use all available evidence.

Our strategy is based on expressed sequences as ESTs, but it can be easily applied to transcriptome sequences generated by next generation sequencing technologies. Indeed, recent tools as Cufflinks [49] can predict alternative transcripts and individual introns, making our methodology extremely recent and useful to improve genome annotations also in absence of canonical ESTs (generally produced by Sanger sequencing).

## Figures and Tables

**Table 1:** Accuracy of gene finding programs on the complete *C. elegans* genome.

Evaluation at gene level																		
Program	SNg	SPg	SSg	WG	MG	SNe	SPe	SSe	WE	ME	SNi	SPi	SSi	WI	MI	SNn	SPn	SSn
GeneID	0.97	0.83	0.90	0.19	0.03	0.67	0.69	0.68	0.16	0.15	0.70	0.74	0.72	0.26	0.30	0.87	0.88	0.87
ASPic	0.34	0.96	0.65	0.03	0.66	0.29	**0.91**	0.60	0.02	0.69	0.30	**0.97**	0.63	0.03	0.70	0.31	**0.98**	0.64
ASPic-GeneID	**0.99**	0.93	0.96	0.09	0.01	0.85	0.81	0.83	0.08	0.03	0.93	0.88	0.90	0.12	0.07	0.95	0.95	**0.95**
ASPic-GeneID_AS1	**0.99**	0.75	0.87	0.21	0.01	**0.87**	0.78	0.82	0.12	0.02	0.93	0.86	0.89	0.14	0.07	**0.96**	0.91	0.93
ASPic-GeneID_AS2	0.98	**0.98**	**0.98**	0.07	0.02	0.86	0.83	**0.84**	0.07	0.03	**0.94**	0.89	**0.91**	0.11	0.06	**0.96**	0.93	0.94
TWINSCAN	0.95	0.87	0.91	0.12	0.05	0.76	0.77	0.76	0.11	0.11	0.81	0.83	0.82	0.17	0.19	0.90	0.91	0.90
TWINSCAN_EST	0.95	0.88	0.91	0.09	0.05	0.79	0.81	0.80	0.09	0.09	0.84	0.87	0.85	0.13	0.16	0.91	0.92	0.91
FGENESH	0.97	0.88	0.92	0.10	0.03	0.76	0.74	0.75	0.13	0.09	0.80	0.79	0.79	0.21	0.20	0.93	0.89	0.91
Genefinder	0.95	0.97	0.96	0.05	0.05	0.77	0.74	0.75	0.13	0.08	0.83	0.78	0.80	0.22	0.17	0.93	0.89	0.91
SNAP	0.96	0.69	0.82	0.22	0.04	0.70	0.66	0.68	0.18	0.12	0.74	0.73	0.73	0.27	0.26	0.90	0.86	0.88

Evaluation at transcript level																		
Program	SNt	SPt	SSt	WT	MT	SNet	SPet	SSet	WEt	MEt	SNit	SPit	SSit	WIt	TMIt	SNnt	SPnt	SSnt

GeneID	0.23	0.23	0.23	0.05	0.19	0.68	0.70	0.69	0.17	0.18	0.70	0.72	0.71	0.28	0.30	0.84	0.87	0.85
ASPic	0.26	**0.71**	**0.48**	0.01	0.29	0.81	**0.95**	**0.88**	0.01	0.15	0.82	**0.98**	**0.90**	0.02	0.18	0.81	**0.99**	0.90
ASPic-GeneID	0.44	0.47	0.45	0.03	0.17	0.86	0.81	0.83	0.10	0.05	0.92	0.85	0.88	0.15	0.08	0.93	0.93	**0.93**
ASPic-GeneID_AS1	**0.53**	0.44	**0.48**	0.08	0.11	0.87	0.85	0.86	0.08	0.06	0.91	0.88	0.89	0.12	0.09	0.92	0.94	**0.93**
ASPic-GeneID_AS2	0.46	0.50	**0.48**	0.03	0.17	**0.88**	0.81	0.84	0.11	0.04	**0.94**	0.85	0.89	0.15	0.06	**0.95**	0.90	0.92
TWINSCAN	0.35	0.36	0.35	0.04	0.15	0.77	0.78	0.77	0.11	0.12	0.81	0.82	0.81	0.18	0.19	0.88	0.91	0.89
TWINSCAN_EST	0.43	0.45	0.44	0.04	0.13	0.80	0.83	0.81	0.08	0.11	0.84	0.87	0.85	0.13	0.16	0.89	0.93	0.91
FGENESH	0.32	0.33	0.32	0.06	0.16	0.75	0.76	0.75	0.13	0.13	0.78	0.79	0.78	0.21	0.22	0.88	0.89	0.88
Genefinder	0.30	0.35	0.32	0.05	0.18	0.79	0.73	0.76	0.16	0.10	0.83	0.75	0.79	0.25	0.17	0.91	0.86	0.88
SNAP	0.27	0.22	0.24	0.09	0.11	0.67	0.73	0.70	0.11	0.19	0.70	0.77	0.73	0.23	0.30	0.82	0.92	0.87

The highest values are shown in bold. SN indicates sensitivity. SP indicates specificity. SS indicates the average between SN and SP. Gene (g), transcript (t), exon (e), intron (i), and nucleotide (n) were assessed.

**Table 2:** Accuracy of gene finding programs on the ENCODE pilot regions.

Evaluation at gene level																		
Program	SNg	SPg	SSg	WG	MG	SNe	SPe	SSe	WE	ME	SNi	SPi	SSi	WI	MI	SNn	SPn	SSn
DOGFISH	0,75	0,84	0,80	0,26	0,25	0,61	0,72	0,67	0,18	0,28	0,61	0,72	0,67	0,28	0,39	0,68	0,80	0,74
Ensembl	0,94	0,81	0,88	0,18	0,06	0,81	0,74	0,78	0,15	0,08	0,87	0,82	0,85	0,18	0,13	0,91	0,86	**0,89**
Exogean	0,83	**0,95**	**0,89**	0,12	0,17	0,87	0,78	0,83	0,12	0,09	**0,90**	0,82	0,86	0,18	0,10	0,85	**0,88**	0,87
Exonhunter	0,94	0,26	0,60	0,72	0,06	0,69	0,41	0,55	0,51	0,13	0,72	0,49	0,61	0,51	0,28	0,90	0,58	0,74
FGENESH++	0,93	0,55	0,74	0,42	0,07	0,78	0,63	0,71	0,31	0,13	0,78	0,67	0,73	0,33	0,22	0,88	0,70	0,79
GeneID	0,92	0,76	0,84	0,32	0,08	0,56	0,59	0,58	0,27	0,28	0,53	0,59	0,56	0,41	0,47	0,77	0,74	0,76
Genemark	0,94	0,43	0,69	0,50	0,06	0,53	0,46	0,50	0,41	0,28	0,51	0,50	0,51	0,50	0,49	0,77	0,61	0,69
PAIRAGON-any	0,91	0,76	0,84	0,23	0,09	0,85	**0,83**	**0,84**	0,14	0,10	0,85	0,86	0,86	0,14	0,15	0,88	0,86	0,87
PAIRAGON-Multiple	0,88	0,80	0,84	0,24	0,12	0,75	0,77	0,76	0,18	0,16	0,75	0,78	0,77	0,22	0,25	0,85	0,83	0,84
SGP2	0,92	0,39	0,66	0,54	0,08	0,65	0,48	0,57	0,40	0,16	0,66	0,55	0,61	0,45	0,34	0,84	0,66	0,75
TWINSCAN	0,89	0,74	0,82	0,30	0,11	0,71	0,58	0,65	0,30	0,17	0,73	0,62	0,68	0,38	0,27	0,86	0,71	0,79
AUGUSTUS-abinitio	0,90	0,57	0,74	0,33	0,10	0,60	0,61	0,61	0,28	0,26	0,57	0,62	0,60	0,38	0,43	0,80	0,71	0,76
AUGUSTUS -any	0,95	0,66	0,81	0,26	0,05	0,81	0,73	0,77	0,21	0,08	0,79	0,74	0,77	0,26	0,21	**0,93**	0,78	0,86
AUGUSTUS-dual	0,93	0,60	0,77	0,29	0,07	0,70	0,65	0,68	0,25	0,15	0,68	0,67	0,68	0,33	0,32	0,89	0,75	0,82
AUGUSTUS_EST	0,95	0,70	0,83	0,24	0,05	0,80	0,74	0,77	0,20	0,09	0,78	0,75	0,77	0,25	0,22	0,91	0,79	0,85
ASPic	0,84	0,66	0,75	0,29	0,16	0,87	0,75	0,81	0,11	0,09	0,89	**0,87**	**0,88**	0,13	0,11	0,81	0,85	0,83
ASPic-GeneID_AS1	0,89	0,54	0,72	0,42	0,11	0,88	0,71	0,80	0,16	0,08	0,89	0,85	0,87	0,15	0,11	0,85	0,72	0,79
ASPic-GeneID_AS2	**0,96**	0,46	0,71	0,50	0,04	**0,90**	0,62	0,76	0,21	0,06	**0,90**	0,76	0,83	0,24	0,10	**0,93**	0,60	0,77
ASPic-GeneID	**0,95**	0,56	0,76	0,46	0,05	0,81	0,65	0,73	0,28	0,05	0,86	0,72	0,79	0,28	0,14	0,89	0,64	0,77

Evaluation at transcript level																		
Program	SNt	SPt	SSt	WT	MT	SNet	SPet	SSet	WEt	MEt	SNit	SPit	SSit	WIt	TMIt	SNnt	SPnt	SSnt

DOGFISH	0,06	0,13	0,10	0,01	0,44	0,73	0,76	0,75	0,15	0,19	0,72	0,75	0,74	0,25	0,28	0,78	0,82	0,80
Ensembl	0,25	0,24	0,25	0,13	0,23	0,84	0,87	0,86	0,04	0,08	0,90	0,93	**0,92**	0,07	0,10	**0,93**	0,95	**0,94**
Exogean	0,51	0,43	0,47	0,26	0,10	**0,89**	0,89	0,89	0,08	0,07	0,90	0,90	0,90	0,10	0,10	0,91	0,91	0,91
Exonhunter	0,06	0,03	0,05	0,03	0,46	0,69	0,67	0,68	0,21	0,19	0,71	0,70	0,71	0,30	0,29	0,85	0,80	0,83
FGENESH++	0,43	0,38	0,41	0,07	0,30	0,80	0,85	0,83	0,08	0,14	0,80	0,86	0,83	0,14	0,20	0,87	0,92	0,90
GeneID	0,03	0,04	0,04	0,01	0,50	0,60	0,62	0,61	0,24	0,27	0,57	0,60	0,59	0,40	0,43	0,78	0,78	0,78
Genemark	0,05	0,04	0,05	0,05	0,41	0,46	0,61	0,54	0,22	0,41	0,45	0,62	0,54	0,38	0,55	0,65	0,79	0,72
PAIRAGON-any	0,51	**0,46**	0,49	0,19	0,21	**0,89**	0,92	**0,91**	0,05	0,08	0,89	0,93	0,91	0,07	0,11	0,90	0,94	0,92
PAIRAGON-Multiple	0,21	0,35	0,28	0,01	0,43	0,87	0,83	0,85	0,12	0,08	0,87	0,83	0,85	0,17	0,13	0,91	0,88	0,90
SGP2	0,05	0,04	0,05	0,06	0,41	0,63	0,66	0,65	0,19	0,23	0,65	0,69	0,67	0,31	0,35	0,76	0,85	0,81
TWINSCAN	0,10	0,08	0,09	0,23	0,22	0,74	0,68	0,71	0,22	0,16	0,76	0,69	0,73	0,31	0,24	0,84	0,78	0,81
AUGUSTUS-abinitio	0,13	0,16	0,15	0,04	0,38	0,59	0,73	0,66	0,15	0,31	0,56	0,71	0,64	0,29	0,44	0,74	0,86	0,80
AUGUSTUS -any	0,27	0,34	0,31	0,04	0,41	0,80	0,86	0,83	0,07	0,14	0,79	0,86	0,83	0,14	0,21	0,88	0,93	0,91
AUGUSTUS-dual	0,15	0,17	0,16	0,05	0,39	0,65	0,77	0,71	0,11	0,25	0,64	0,77	0,71	0,23	0,36	0,79	0,90	0,85
AUGUSTUS_EST	0,27	0,36	0,32	0,03	0,42	0,80	0,86	0,83	0,07	0,14	0,79	0,86	0,83	0,14	0,21	0,88	0,94	0,91
ASPic	0,63	0,37	**0,50**	0,37	0,06	0,84	**0,95**	0,90	0,02	0,13	0,85	**0,96**	0,91	0,04	0,15	0,86	**0,97**	0,92
ASPic-GeneID_AS1	**0,65**	0,33	0,49	0,33	0,06	0,84	0,94	0,89	0,02	0,13	0,85	**0,96**	0,91	0,04	0,15	0,86	**0,97**	0,92
ASPic-GeneID_AS2	0,64	0,25	0,45	0,41	0,05	0,84	0,93	0,89	0,03	0,12	0,85	0,94	0,90	0,06	0,15	0,88	0,96	0,92
ASPic-GeneID	0,21	0,22	0,22	0,02	0,48	0,86	0,77	0,82	0,15	0,06	**0,91**	0,81	0,86	0,19	0,09	0,91	0,85	0,88

The highest values are shown in bold. SN indicates sensitivity. SP indicates specificity. SS indicates the average between SN and SP. Gene (g), transcript (t), exon (e), intron (i), and nucleotide (n) were assessed.
